# Human umbilical cord and dental pulp-derived mesenchymal stem cells: Biological characteristics and potential roles *in vitro* and *in vivo*

**DOI:** 10.3892/mmr.2015.3198

**Published:** 2015-01-14

**Authors:** XIAOYAN CUI, LEI CHEN, TING XUE, JING YU, JIE LIU, YAZHONG JI, LIMING CHENG

**Affiliations:** 1 Translational Center for Stem Cell Research, Tongji Hospital, Tongji University School of Medicine, Shanghai 200065, P.R. China; 2 Department of Spine Surgery, Tongji Hospital, Tongji University School of Medicine, Shanghai 200065, P.R. China; 3 Department of Reproductive Medicine, Tongji Hospital, Tongji University School of Medicine, Shanghai 200065, P.R. China

**Keywords:** human umbilical cord-derived mesenchymal stem cells, survivin, dental pulp-derived stem cells, differentiation, RNA sequencing

## Abstract

Mesenchymal stem/stromal cells (MSCs) have a wide application in cell-based therapies and tissue engineering. In the present study, the differentiation, survivin (SVV)-modified effects and molecular basis of human umbilical cord-derived MSCs (HUMSCs) and dental pulp-derived stem cells (DPSCs) were investigated. The HUMSCs were found to differentiate into adipocytes more readily than the DPSCs and the HUMSCs and DPSCs were each able to differentiate into osteoblasts and chondroblasts. Following modification of the MSCs by SVV, the secretion of SVV in the modified HUMSCs was significantly higher compared with that in the modified DPSCs. *In vivo*, survival of the SVV-modified DPSCs was observed at 4 and 14 days after intrastriatal transplantation, as was the expression of SVV and differentiation into astrocytes. The gene expression profiles of the control and modified HUMSCs and DPSCs were compared using RNA sequencing and an association was observed between gene expression and variability in cell line function. These findings provide novel information regarding the differences between HUMSCs and DPSCs and insight into optimal cell sources for therapeutic applications.

## Introduction

Mesenchymal stem/stromal cells (MSCs) are able to extensively self renew and possess a multilineage differentiation potential (1–3). In addition, MSCs exert a supportive function through paracrine effects (4–6). Therefore, these cells have a wide application in cell-based therapies and tissue engineering. Over the last decade, MSCs derived from alternative and easily accessible sources have received increasing attention. MSCs can be isolated from a large variety of fetal tissues, extraembryonic tissues and organs and a variety of tissues from children and adults (7).

Human umbilical cord-derived MSCs (HUMSCs) can be isolated and expanded easily *in vitro* without ethical concerns. Therefore, they have received interest as a promising candidate for several potential clinical applications (8) and several studies have demonstrated that HUMSCs exhibit this potential (9). HUMSCs may possess a capacity for autologous and allogenic transplantation due to their immunoprivileged properties (10). They are able to effectively suppress mitogen-induced T-cell proliferation (11) and low levels of rejection have been observed in animal transplantation studies (12,13).

HUMSCs and dental pulp-derived stem cells (DPSCs) offer available sources of autologous cells without ethical controversy. Human DPSCs were initially reported by Gronthos *et al* in 2000 and were observed to share a similar morphology and *in vitro* differentiation potential with bone marrow MSCs (14). DPSCs also possess immunoregulatory properties and are capable of inducing activated T-cell apoptosis *in vitro* and alleviating inflammatory-related tissue injury (15). Impacted adult wisdom teeth and naturally-exfoliated deciduous teeth do not ordinarily serve a function in adults. Therefore, DPSCs can be obtained without adverse effects on health.

HUMSCs are also amenable to genetic modification. Kermani *et al* (16) transfected HUMSCs with a green fluorescent protein (GFP)-reporter gene and established a stable cell line. There have been no reports on the genetic modification of DPSCs to date, to the best of our knowledge. Survivin (SVV), a 16.5 kDa protein, is a member of the X-linked inhibitor of apoptosis family and exhibits broadly cytoprotective properties (17). In the present study, SVV-modified HUMSCs and DPSCs were investigated.

The potential clinical application of HUMSCs and DPSCs remains to be elucidated. In the present study, the differentiation, SVV-modified effects and molecular basis of the heterogeneity between HUMSCs and DPSCs were investigated *in vitro* and *in vivo*.

## Materials and methods

### Preparation of HUMSCs and DPSCs

HUMSCs were isolated, as previously described, with certain modifications (18). The human umbilical cords were obtained from babies delivered at full-term following institutional review board approval. A segment of umbilical cord, cut to ~7 cm in length was obtained. This segment was placed immediately into 25 ml complete medium, comprising Dulbecco’s modified Eagle’s medium (DMEM)/F12 (Gibco Life Technologies, Darmstadt, Germany) supplemented with 10% fetal bovine serum (FBS; Gibco Life Technologies, Grand Island, NY, USA), 100 U/ml penicillin and 100 μg/ml streptomycin (Gibco Life Technologies). The tubes were stored on ice for dissection within 4 h. The umbilical cord segment was washed three times with phosphate-buffered saline (PBS) without calcium and magnesium (HyClone, Shanghai, China) and dissected longitudinally using an aseptic technique. The umbilical vein and the umbilical arteries were removed and the umbilical cord segment was cut into a 0.5–1 mm^3^ tissue block and incubated in 3 ml 0.2% collagenase type II (Sigma-Aldrich, St. Louis, MO, USA) for 1 h at 37°C. A five-fold volume of complete medium was added and the supernatant was filtered using a 70-μm filter following free settling for 20 min. The cells were collected following centrifugation at 500 × g for 5 min and plated in plastic culture flasks at a concentration of 5×10^3^/cm^2^. After 3 days, the non-adherent cells were removed by washing three times with PBS. The medium was changed every 2–3 days.

The human DPSCs were isolated as follows: Two adult wisdom teeth from one patient aged between 18 and 30 years old, which were extracted for clinical purposes at Tonji Hospital, Tonji University School of Medicine (Shanghai, China), were collected and the crown and root were separated. The dental pulp was isolated and digested in 0.3% collagenase type I (Sigma-Aldrich) for 1 h at 37°C. The DPSCs were cultured in α-minimum essential medium (α-MEM; HyClone) supplemented with 10% FBS at 37°C in 5% CO_2_.

The HUMSCs and DPSCs were identified according to the expression of antigens using flow cytometry with a Human Mesenchymal Stem Cell Functional Identification kit (BD Biosciences, San Jose, CA, USA).

The study was approved by the ethics committee of Tonji Hospital, Tonji University School of Medicine (Shanghai, China). Written informed consent was obtained from all patients or their families.

### Differentiation characterization

The adipogenic, osteogenic and chondrogenic differentiation potentials of the HUMSCs and DPSCs were determined by incubating the cells in differentiation media using a Human Mesenchymal Stem Cell Functional Identification kit (R&D Systems, Inc, Minneapolis, MN, USA).

### Adipogenic differentiation

The HUMSCs and DPSCs were seeded onto 12 mm cover slips at a density of 3.7×10^4^ cells/well in the α-MEM basal medium supplemented with 10% FBS, 100 U/ml penicillin, 100 μg/ml streptomycin and 2 mM L-glutamine. Theells were cultured in an incubator at 37°C and 5% CO_2_. At 100% confluence, the medium was replaced with the adipogenic differentiation medium containing hydrocortisone, isobutylmethylxanthine, indomethacin in 95% ethanol and α minimum essential medium basal medium. (R&D Systems, Inc). The medium was replaced every 3 days. After 2 weeks, the cells on the cover slips were washed with PBS twice and fixed with 4% paraformaldehyde (Beijing Solarbio Science & Technology, Beijing, China) for 20 min at room temperature. The cells were then washed with PBS and stained for 30 min using oil red O (Sigma-Aldrich). Following washing twice with PBS, images of cells were captured under a phase contrast microscope (IX71, Olympus Corp., Tokyo Japan).

### Osteogenic differentiation

In order to induce osteogenic differentiation, 7.4×10^3^ HUMSCs and DPSCs per well were cultured for 3 weeks in α-MEM basal medium supplemented with 10% FBS, 100 U/ml penicillin, 100 μg/ml streptomycin and 2 mM L-glutamine (Gibco Life Technologies). At 50–70% confluency, the medium was replaced with the osteogenic differentiation medium (R&D Systems, Inc), which was replaced every 3 days. The cells were fixed with 4% paraformaldehyde for 20 min, washed with PBS and stained using alizarin red S (Sigma-Aldrich). Images of the cells were then captured under a phase contrast microscope (IX71; Olympus Corp.).

### Chondrogenic differentiation

In order to induce chondrogenic differentiation, 2.5×10^5^ cells were centrifuged at 200 × g for 5 min at room temperature and resuspended in DMEM/F-12 basal medium supplemented with 0.5 ml 100X ITS supplement (R&D Systems, Inc), 100 U/ml penicillin, 100 μg/ml streptomycin and 2 mM L-glutamine. The cells were then centrifuged at 200 × g for 5 min, resuspended in chondrogenic differentiation medium (R&D Systems, Inc) and centrifuged again. The resulting pellets were incubated at 37°C and 5% CO_2_ for 3 weeks and the medium was replaced every 3 days. The pellets were fixed with 4% paraformaldehyde in PBS for 20 min at room temperature and then frozen and sectioned using standard cryosectioning methods. The sections were stained using polyclonal goat anti-human aggrecan antibody (1:10; #962644) and NorthernLights 557-fluorochrome-conjugated donkey anti-goat secondary antibody (1:200; #NL001; R&D Systems, Inc) and images were captured under a fluorescence microscope.

### Lentiviral vectors and MSC transduction

The MSCs were transduced with a pLVX-IRES-ZsGreen1 vector (Clontech, Mountain View, CA, USA) containing an inserted full length cDNA of SVV. Vectors without this insertion were used as a control. The volume of lentivirus used for each transduction was determined by titration as the volume required to generate 90–95% ZsGreen1-positive MSCs after 3 days.

### Measurement of the levels of SVV in the cell culture supernatant

The transduced MSCs were plated in six-well plates (5,000 cells/cm^2^) overnight at 37°C in 5% CO_2_. The medium was then replaced with 2 ml/well DMEM/F12 for the HUMSCs and α-MEM for the DPSCs, followed by incubation for 48 h at 37°C in 5% CO_2_. The supernatants were collected in order to confirm overexpression and secretion of SVV using a human enzyme-linked immunosorbent assay (ELISA) kit according to the manufacturer’s instructions (Abcam, Cambridge, MA, USA). The cell number was determined for normalization.

### Western blotting

To detect the overexpression of SVV in the HUMSCs and DPSCs, the proteins in the transduced cells were extracted using radioimmunoprecipitation assay lysis buffer supplemented with 1 mM phenylmethylsulfonyl fluoride (Beyotime Institute of Biotechnology, Haimen, China). The proteins were loaded onto 12% SDS-PAGE gels and transferred to polyvinyline difluoride membranes (EMD Millipore, Billerica, MA, USA). Following blocking with 5% defatted milk [defatted milk powder (Quechao, Beijing, China) in tris-buffered saline supplemented with Tween20] for 1 h, the membranes were incubated with survivin (FL-142) rabbit polyclonal antibody raised against amino acids 1-142 of human survivin (1:100; sc-10811; Santa Cruz Biotechnology, Inc., Santa Cruz, CA, USA) overnight at 4°C.

### DPSC transplantation

As been previous studies have examined the survival and differentiation of umbilical cord-derived MSCs *in vivo* (19), the current study focused on the properties of the DPSCs *in vivo*.

All experimental procedures involving animals were conducted under the approval of the Animal Care and Use Committee of Tongji University (Shanghai, China). Female C57BL/6 mice were housed at 22°C and 65% humidity with a 12 h light/dark cycle and access to food and water *ad libitum*.

The mice were randomly assigned to one of the following three groups: Control mice injected with PBS (n=6); mice transplanted with DPSCs (n=6) and mice transplanted with SVV-modified DPSCs (n=6). The adult 8-week-old mice were anesthetized using 0.5% pentobarbital sodium (Sigma-Aldrich). The heads of the mice were shaved and cleaned and the mice were then positioned in the stereotaxic apparatus, with anesthesia maintained during surgery. A midline incision was made on the scalp and the skin was retracted to expose the bregma. Burr holes (0.5 mm) were then placed directly over the striatum (coordinates: anterior/posterior, +0.5 mm; medial/lateral, +2.0 mm from the bregma). The control and SVV-modified DPSCs were resuspended at a density of 100,000 cells/μl in PBS. The cells were transplanted (−3.5 mm ventral to the skull surface) into the control and SVV-modified DPSCs at a constant rate of 0.5 μl/min in a total injection volume of 2 μl/mouse using a 10-μl Hamilton microsyringe (Dalian Replete Scientific Instrument Co. Ltd, Dalian, Liaoning, China). The syringe was left in place for 3 mins and then retracted. The wound was closed and the mice were placed on heat insulation pads (Kent Scientific Corporation, Torrington, CT, USA) until conscious.

### Immunocytochemistry

Immunocytochemistry was performed in order to examine the survival and differentiation of the control and SVV-modified DPSCs in the brains of the mice. Following cell transplantation, the mice were anesthetized for either 4 or 14 days using pentobarbital sodium and perfused transcardially with 0.1 M PBS, followed by 4% paraformaldehyde diluted in 0.1 M PBS (pH 7.4) to fix the tissues. The brains were removed promptly, post-fixed in 4% paraformaldehyde overnight at 4°C and then transferred to 30% sucrose in 0.1 M PBS for 2 days at 4°C. Coronal sections (20 μm) were then cut and mounted on positively-charged microscope slides (Ruihoge, Nanjing, China). For immunohistochemical analysis, the tissue was blocked using 10% normal goat serum (CST, Boston, MA, USA) in PBS containing 0.1% Triton-X (Sigma-Aldrich) for 45 min at room temperature. The tissues were then cultured with primary antibodies against rabbit SVV (1:100; sc-10811; Santa Cruz Biotechnology, Inc.), rabbit anti-human/mouse/rat neuronal nuclei (NeuN; 1:500; 1:500; Abcam) and rabbit anti-cat/dog/mouse/rat/sheep glial fibrillary acidic protein (GFAP; 1:500; Nr.z 0334; Dako North America, Inc. Carpinteria, CA, USA) at 4°C overnight. All antibodies were polyclonal. The following day, the tissues were rinsed three times in PBS with 1% bovine serum albumin and incubated with conjugated secondary antibodies (goat anti-rabbit AlexaFluor594; 1:500; A-11037; Invitrogen Life Technologies) for 1 h at room temperature. Subsequently, 4′,6-diamidino-2-phenylindole (1:1,000; Sigma-Aldrich) was used to label the positions of the cell nuclei. Images of the fluorescent labels were captured using a Nikon fluorescent microscope (Eclipse Ti-S; Nikon Corporation, Tokyo, Japan) at ×20 magnification. All the images were analyzed using ImageJ software (National Institutes of Health; Bethesda, MD, USA). Briefly, images of the transplanted cells were captured from five sections of each animal and the average intensity of the labels in each group was analyzed.

### RNA sequencing and analysis

In order to compare the gene expression profile between the control and the SVV-modified HUMSCs and DPSCs, RNA isolation was performed on the cells using TRIzol^®^ reagent (Invitrogen Life Technologies) and the total RNA was then digested with DNase I (Invitrogen Life Technologies) to ensure that the samples were not contaminated with genomic DNA. Duplicate experiments were performed for each sample. Library preparation and paired end sequencing (101 bp length) were performed using an Illumina HiSeq 2500 sequencer (Illumina, San Diego, CA, USA). Short reads were aligned to the reference human genome (*Homo sapiens* GRCh37) using TopHat (20). The gene annotations were from the Ensembl database, packaged using iGenome (Illumina). The sequence reads were used to calculate the overall gene expression in terms of the reads per kilobase of exon per million mapped reads (RPKM). The differentially expressed transcripts were screened using the cufflinks procedure (21).

### Abundant genes

Genes with an RPKM value >100 and levels of gene expression ≥2-fold higher than the control groups were identified in the HUMSCs, DPSCs and SVV-modified HUMSCs and DPSCs and functional enrichment analyses were performed using the Gene Ontology database (http://www.geneontology.org/GO.downloads.annotations.shtml). and the Kyoto Encyclopedia of Genes and Genomes pathway database (http://www.genome.jp/kegg/pathway.html).

### Genes associated with stem cell differentiation

A total of five genes associated with stem cell differentiation, with RPKM values >5 in the control and SVV-modified HUMSCs and DPSCs, were selected based on the Gene Ontology database.

### Cytokine-cytokine receptor interaction genes

A total of eight genes associated with cytokine-cytokine receptor interactions, with RPKM values >5 in the control and SVV-modified HUMSCs and DPSCs, were selected based on the Kyoto Encyclopedia of Genes and Genomes pathway database.

### Data presentation and statistical analysis

Data are presented as the mean ± standard error of the mean (error bars). All significant differences were evaluated using Student’s t-test or analysis of variance. Statistical analyses were performed using SPSS 15.0 (SPSS, Inc., Chicago, IL, USA). P<0.05 was considered to indicate a statistically significant difference.

## Results

### Phenotypic characterization of isolated HUMSCs and DPSCs

**Figure 1:**
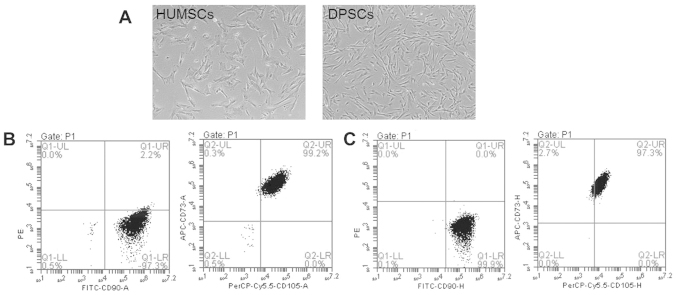
Phenotypic characterization of HUMSCs and DPSCs. (A) Passage 5 DPSCs were more similar to fibroblasts than the HUMSCs. Scale bar: 500 μm. (B) 96.5% of the HUMSCs were positive for CD73, CD90 and CD105, but negative for CD34, CD45, CD11b or CD14, CD19, CD79α and HLA-DR. (C) 99.2% of the DPSCs were positive for CD73, CD90 and CD105, but negative for CD34, CD45, CD11b or CD14, CD19, CD79α and HLA-DR. HUMSCs, human umbilical cord-derived mesenchymal stem cells; DPSCs, dental pulp-derived stem cells; FITC, fluorescein isothiocyanate; CD, cluster of differentiation.

The shapes of the DPSCs were more similar to those of fibroblasts compared with the HUMSCs (Fig. 1A). Flow cytometric analysis revealed that the HUMSCs and DPSCs expressed a set of cluster of differentiation (CD) MSC markers (CD90, CD73 and CD105), however, they did not express endothelial/hematopoietic markers (CD34, CD45, CD11b or CD14, CD19 or CD79α or HLA-DR; Fig. 1B and C).

### Identification of differentiation

**Figure 2:**
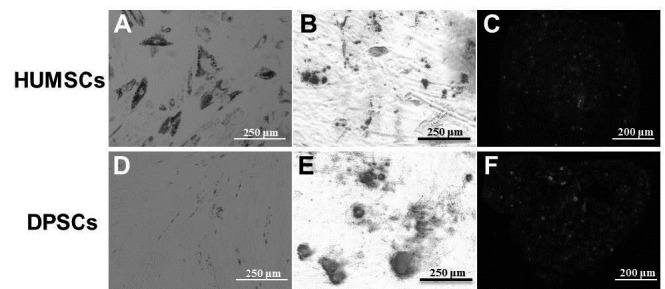
Adipogenic, osteogenic and chondrogenic differentiation of the (A-C) HUMSCs and (D-F) DPSCs, respectively. Lipid droplets, stained using oil red O, were present in the (A) HUMSCs, but not the (D) DPSCs (Scale bar: 250 μm). Osteogenic differentiation in the (B) HUMSCs and (E) DPSCs was detected using alizarin red S staining of calcium precipitation (Scale bar, 250 μm). Aggrecan, a chondrogenic marker, was highly expressed in the (C) HUMSCs and (F) DPSCs (Scale bar, 200 μm). HUMSCs, human umbilical cord-derived mesenchymal stem cells; DPSCs, dental pulp-derived stem cells.

Following induction with adipogenic differentiation medium for 2 weeks, lipid droplets, stained using oil red O, were observed in the HUMSCs (Fig. 2A), but not the DPSCs (Fig. 2D). For osteogenic differentiation, the cells were cultured for 3 weeks in osteogenic media. Calcium precipitation, determined by alizarin red S staining, was observed in the HUMSCs and the DPSCs (Fig. 2B and E). The chondrogenic differentiation capacity of the HUMSCs and DPSCs were evaluated by examining the expression of aggrecan, a chondrogenic marker. Following culture with the HUMSCs and DPSCs in chondrogenic differentiation medium for 3 weeks, the cells exhibited increased expression of aggrecan (Fig. 2C and F).

### Efficiency of MSC transduction with lentiviral vectors containing SVV

**Figure 3:**
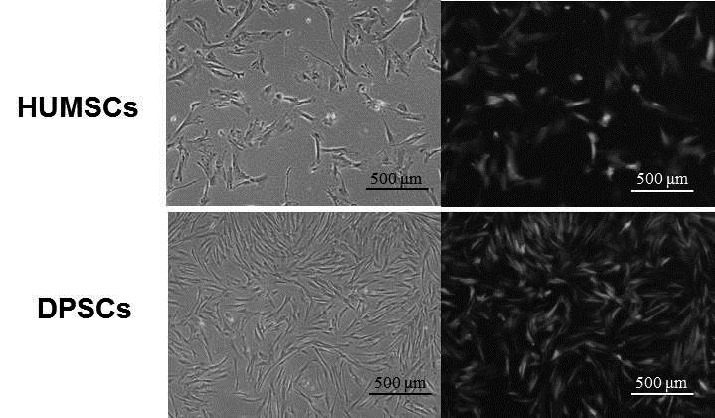
Gene transduction. The MSCs were transduced with a pLVX-IRES-ZsGreen1 vector containing an inserted full length cDNA of SVV. Scale bar, 500 μm. Expression of green fluorescent protein in the SVV-modified MSCs indicated that the efficiency of gene transduction was >90%. MSCs, mesenchymal stem cells; HUMSCs, human umbilical cord-derived MSCs; DPSCs, dental pulp-derived stem cells; SVV, survivin.

Following infection with the SVV recombinant lentivirus, overexpression of GFP was observed in the HUMSCs and DPSCs (Fig. 3). The efficiency of gene transduction was >90%.

### Protein expression of SVV

**Figure 4:**
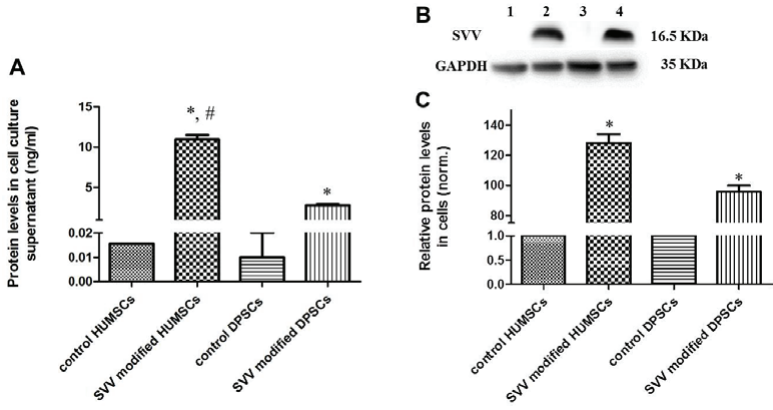
Protein levels of SVV are significantly increased in the SVV-modified HUMSCs and DPSCs. (A) Protein levels of SVV were measured in the cell culture supernatant using an enzyme-linked immunosorbent assay. (B) Representative western blot analysis of the control, SVV-modified HUMSCs and DPSCs. The level of GAPDH was used as an internal control. Lanes: 1, HUMSCs; 2, SVV-modified HUMSCs; 3, DPSCs; 4, SVV-modified DPSCs. (C) Quantitative analysis revealed that the modified HUMSCs and DPSCs had a significantly higher expression of SVV. (^*^P<0.05, compared with the control and ^#^P<0.05, compared with the SVV-modified DPSCs. HUMSCs, human umbilical cord-derived mesechymal stem cells; DPSCs, dental pulp-derived stem cells; SVV, survivin.

The protein expression levels of SVV were determined by ELISA and western blotting. The protein levels of SVV were significantly increased in the cell culture supernatant of the SVV-modified HUMSCs and DPSCs compared with levels in the control groups (Fig. 4A). Furthermore, the secretion of SVV in the modified HUMSCs was significantly higher compared with that observed in the modified DPSCs. The western blotting revealed that modified HUMSCs and DPSCs exhibited a significant increase in the expression of SVV (Fig. 4B and C).

### Control and SVV-modified DPSCs survive following intrastriatal transplantation

**Figure 5:**
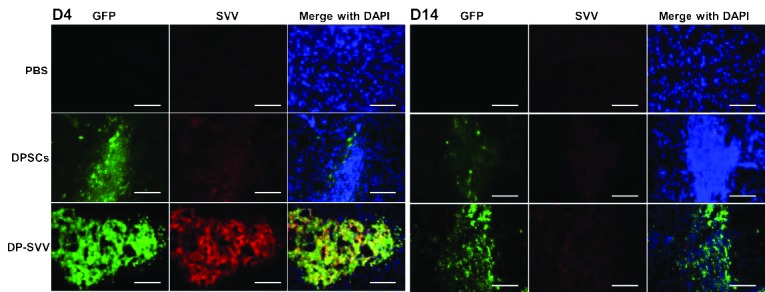
Survival of the control and DP-SVV cells in the wild-type C57B/6 mice 4 and 14 days after transplantation. Microscopic analysis of the immunofluorescence-labeled striatal sections in the cell-transplanted mice. Transplanted cells expressing GFP (green) were immunostained against SVV (red) and counterstained with DAPI (blue). Scale bar, 100 μm. DPSCs, dental pulp-derived stem cells; SVV, survivin; DP-SVV, SVV-modified DPSCs; PBS, phosphate-buffered saline; GFP, green fluorescent protein; DAPI, 4′,6-diamidino-2-phenylindole.

**Figure 6:**
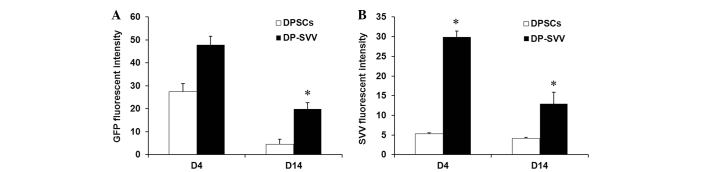
Quantification of GFP and SVV fluorescence intensity of the transplanted control DPSCs and DP-SVV cells in the striata 4 and 14 days after transplantation. (A) GFP fluorescence intensity was not significantly different between the DPSCs and DP-SVV on day 4, but was decreased on day 14. The GFP fluorescence intensity of DP-SVV was more marked compared with that of the control DPSCs. (B) SVV fluorescence intensity of the DP-SVV cells was more marked compared with that of the control DPSCs on days 4 and 14. ^*^P<0.05, compared with the control DPSCs. DPSCs, dental pulp-derived stem cells; SVV, survivin; DP-SVV, SVV-modified DPSCs; GFP, green fluorescent protein.

In the present study, the survival and the expression of SVV in the control and SVV-modified DPSCs were investigated *in vivo*. The results demonstrated that the GFP signal, expressed by the inserted pLVX-IRES-ZsGreen1 vector, was marked in the control and SVV-modified cell lines 4 days after transplantation, however, it had weakened by day 14 (Fig. 5). In addition, the GFP signal in SVV-modified DPSCs was more marked compared with that in the control cells (Fig. 6). These results suggested that SVV promoted the survival of the DPSCs *in vivo*. The expression of SVV in the transplanted SVV-modified DPSCs was higher than that in the control DPSCs on day 4. However, the expression of SVV in the transplanted DPSCs decreased 14 days after transplantation.

**Figure 7:**
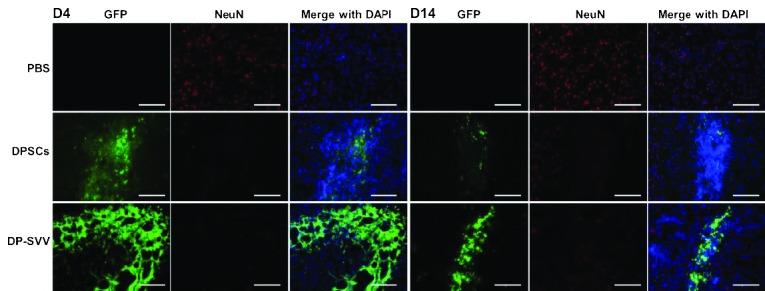
Immunohistochemical analysis of the control and DP-SVV cells using NeuN. Microscopic images revealed no GFP-labeled cells coexpressing NeuN (red) in the striatum at days 4 or 14 post-transplantation. Scale bar, 100 μm. DPSCs, dental pulp-derived stem cells; SVV, survivin; DP-SVV, SVV-modified DPSCs; PBS, phosphate-buffered saline; GFP, green fluorescent protein; NeuN, neuronal nuclei.

**Figure 8:**
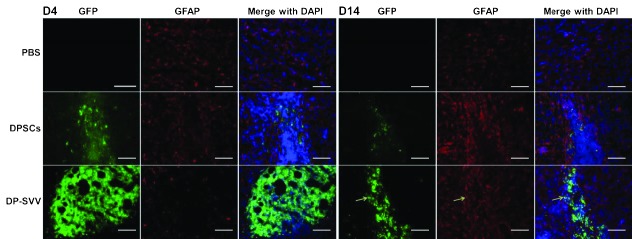
Immunohistochemical analysis of control and DP-SVV cells using GFAP. Microscopic images revealed a few GFP-labeled cells coexpressing GFAP (red) in the DP-SVV group 14 days after transplantation. Scale bar, 100 μm. DPSCs, dental pulp-derived stem cells; SVV, survivin; DP-SVV, SVV-modified DPSCs; GFAP, glial fibrillary acidic protein; PBS, phosphate-buffered saline; GFP, green fluorescent protein.

In addition, the present study examined the differentiation capacity of the control and SVV-modified DPSCs *in vivo*. Neither NeuN (a marker for neurons) nor glial fibrillary acidic protein (GFAP; a marker for astrocytes) were detected in the control or SVV-modified DPSCs 4 days after transplantation (Figs. 7 and 8). However, 14 days after transplantation, a few GFP-labeled cells coexpressed GFAP (Fig. 8), but not NeuN (Fig. 7) in the striatum.

### Gene expression data analysis

**Figure 9:**
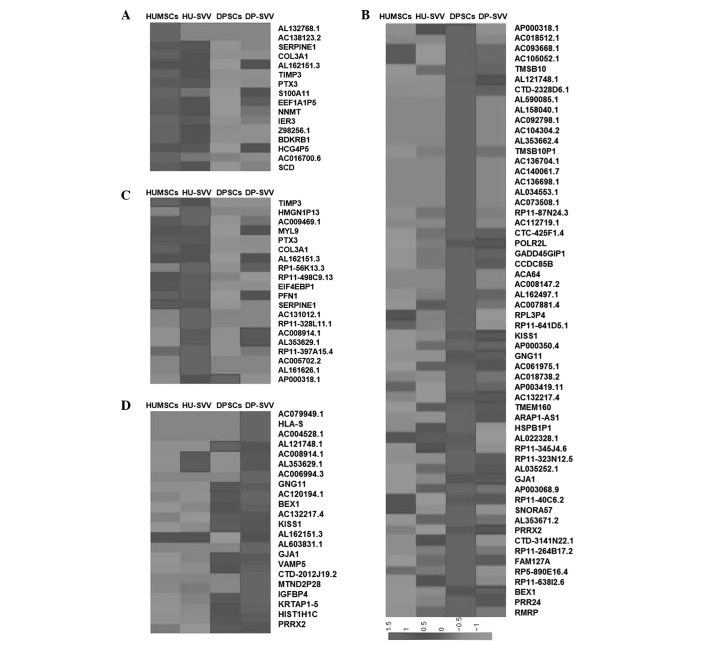
Heat map of group-specific highly expressed genes with RPKM values >100. (A) HUMSC-specific high expression genes. (B) HU-SVV-specific highly expressed genes. (C) DPSC-specific highly expressed genes. (D) DP-SVV-specific highly expressed genes. RPKM, reads per kilobase of exon per million mapped reads; HUMSCs, human umbilical cord-derived mesenchymal stem cells; DPSCs, dental pulp-derived stem cells; SVV, survivin; HU-SVV, SVV-modified HUMScs; DP-SVV, SVV-modified DPSCs.

The heat map of the abundant gene set is shown in Fig. 9. The genes with RPKM values >100 were analyzed with respect to each MSC group. A total of 16, 20, 58 and 22 genes were identified with RPKM values >100 and gene expression levels ≥2-fold higher in the HUMSCs, SVV-modified HUMSCs, DPSCs and SVV-modified DPSCs, respectively, compared with the control (Fig. 9). The most significant biological processes were ‘inflammatory response’, ‘vascular transport’, ‘platelet activation’ and ‘vascular transport’ for the HUMSCs, DPSCs, SVV-modified HUMSCs and SVV-modified DPSCs, respectively. The most significant pathways were ‘complement and coagulation cascades’, ‘hypoxia-inducible factor-1 signaling pathway’, ‘RNA polymerase’ and ‘SNARE interactions in vesicular transport’ for the HUMSCs, DPSCs, SVV-modified HUMSCs and SVV-modified DPSCs, respectively.

**Figure 10:**
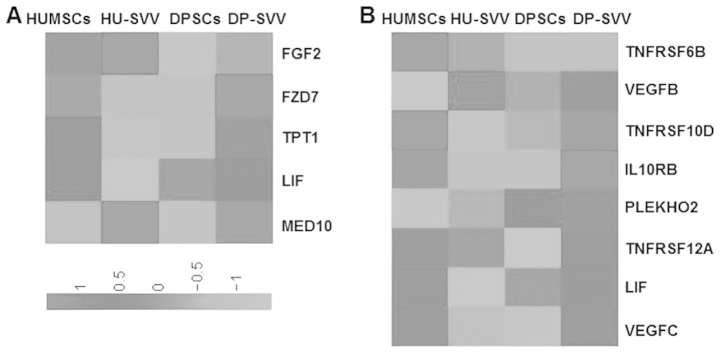
Heat map of genes associated with stem cell differentiation and cytokine-cytokine receptor interaction. (A) Genes associated with stem cell differentiation with RPKM values >5 were compared through RPKM value analysis in the HUMSCs, HU-SVV, DPSCs and DP-SVV cells. (B) Genes associated wirth cytokine-cytokine receptor interactions with RPKM values >5 in the four cell lines. RPKM, reads per kilobase of exon per million mapped reads; HUMSCs, human umbilical cord-derived mesenchymal stem cells; DPSCs, dental pulp-derived stem cells; SVV, survivin; HU-SVV, SVV-modified HUMScs; DP-SVV, SVV-modified DPSCs; TNFRSF, tumor necrosis factor receptor superfamily; VEGF, vascular endothelial growth factor.

The expression of genes associated with biological activities among the groups was analyzed in order to clarify the molecular basis of the heterogeneity. Since MSCs possess multilineage differentiation potential, genes associated with stem cell differentiation were analyzed in the control and the SVV-modified HUMSCs and DPSCs. A total of 128 genes were identified as associated with stem cell differentiation in the four cell lines and genes with RPKM values >5 were selected. Analysis demonstrated higher expression levels of the FGF2 and MED10 stem cell differentiation genes in the SVV-modified HUMSCs, compared with the HUMSCs, DPSCs and SVV-modified DPSCs (Fig. 10A) and increased expression of FZD7 in the SVV-modified DPSCs compared with HUMSCs, HU-SVV and DPSCs. MSCs exhibit anti-inflammatory and immunomodulatory properties (22). Therefore, a comparative analysis of genes associated with cytokine-cytokine receptor interactions in the control and SVV-modified HUMSCs and DPSCs was also performed. A total of 196 genes associated with cytokine-cytokine receptor interactions were identified, from which genes with RPKM values >5 were selected. Clustering highlighted a group of genes with increased expression in the HUMSCs compared with HU-SVV, DPSCs and DP-SVV, including tumor necrosis factor receptor superfamily (TNFRSF)6B, TNFRSF10D, LIF and vascular endothelial growth factor C (VEGFC; Fig. 10B). Clustering also highlighted a group of inflammatory-associated genes, the expression levels of which were highest in the SVV-modified DPSCs, including IL10RB, PLEKHO2, TNFRSF12A and VEGFC.

## Discussion

MSCs may be isolated from a variety of sources, including bone marrow, umbilical cord, adipose tissue and dental pulp, and they may be used in several cell-based therapies and tissue engineering approaches. It is important to select the type of MSCs that are the most promising candidates for specific clinical applications. In the present study, the appearance of the DPSCs were more elongated compared with the HUMSCs. The surface antigens, CD90, CD73 and CD105, were used for the identification of HUMSCs and DPSCs, as these markers are indicated by the International Society for Cellular Therapy as positive markers for human MSCs (23). These MSCs lack expression of surface markers of endothelial or hematopoietic origin, including CD34, CD45, CD11b or CD14, CD19 or CD79α and HLA-DR.

In the present study, lipid droplets were observed in the HUMSCs rather than the DPSCs following the induction of adipogenic differentiation. Osteogenic differentiation was observed through calcium precipitation in the HUMSCs and DPSCs. The chondrogenic differentiation capacity of the HUMSCs and DPSCs was also evaluated by examining the expression level of aggrecan, which was marked in the two cell lines. These results indicated that the HUMSCs were more likely to differentiate into adipocytes compared with the DPSCs and that the two cell types are able to differentiate into osteoblasts and chondroblasts. These findings are consistent with those of Mangano *et al* (14) and Gronthos *et al* (24), which demonstrated that DPSCs can be induced to differentiate into osteoblasts, but not adipocytes.

Following transduction of the HUMSCs and DPSCs with a lentiviral vector, containing the full-length cDNA of survivin insertion, the expression of GFP indicated that the efficiency of gene transduction was >90%. The protein expression of SVV in the HUMSCs and DPSCs was compared using an ELISA assay and western blotting. The protein levels of SVV significantly increased in the SVV-modified HUMSCs and DPSCs compared with the controls. However, the expression of SVV in the cell culture supernatant was significantly higher in the SVV-modified HUMSCs compared with the SVV-modified DPSCs. Therefore, modified HUMSCs secreted more SVV protein compared with the modified DPSCs.

SVV is an important protein for cell death resistance and has been reported to modulate stem cell proliferation (25). As MSCs promote tissue repair by stimulating and modulating tissue-specific cells (4), SVV may enhance these stimulating and modulating effects of MSCs. A previous study on the differentiation of umbilical cord-derived MSCs *in vivo* (19), observed no neuronal or glial differentiation in the transplanted umbilical cord-derived MSCs. Therefore, the present study focused on the survival and differentiation of DPSCs *in vivo*. The results confirmed that SVV promoted the survival and astrocyte differentiation of the DPSCs *in vivo*.

The RNA sequencing analyses provided novel insight into the variable biological properties among the four groups. Higher levels of the expression of FGF2, which is involved in the development of the limb and nervous system, were observed in the SVV-modified HUMSCs and FZD7, which encodes receptors for Wnt signaling proteins, in the SVV-modified DPSCs. Genetic analyses elucidates the MSC-mediated anti-inflammatory and immunomodulatory properties of the different groups. The expression of IL10RB, an anti-inflammatory and immunoregulatory gene, was higher in the SVV-modified DPSCs than in the DPSCs, HUMSCs and SVV-modified HUMSCs. The expression levels of the TNFRSF6B and TNFRSF10D anti-inflammatory genes were higher in the HUMSCs than in DPSCs or SVV-modified DPSCs and HUMSCs. Therefore, HUMSCs express higher levels of anti-inflammatory, immunoregulatory and stem cell differentiation-associated genes than DPSCs.

In conclusion, the source of MSCs and their subsequent modification can be selected according to the difference in differentiation, the effect of genetic modification and the molecular basis of the HUMSCs and DPSCs. However, further investigation is required in order to decide which cell line is most suitable for a specific clinical application.
